# Surgical Outcome of Tethered Cord Syndrome Following Duplicate Filum Terminale Externum Resection in a Patient With Normal Conus Medullaris and Fatty Filum: A Case Report

**DOI:** 10.7759/cureus.93406

**Published:** 2025-09-28

**Authors:** Vanni Veronesi, Camilla Mencarani, Gianluca Bizzocchi, Carlo Sacco

**Affiliations:** 1 Department of Neurosurgery, Local Health Authority of Romagna "Degli Infermi" Hospital, Faenza, ITA; 2 Department of Neuroscience, Local Health Authority of Romagna "Degli Infermi" Hospital, Faenza, ITA

**Keywords:** duplicate filum terminale externum, fatty filum, filum terminale internum, tethered cord syndrome, transhiatal approach

## Abstract

We describe the case of a 38-year-old woman who limped due to a forced antalgic posture involving plantar flexion of the left foot. She exhibited hypoesthesia on the posterior aspect of the left lower limb. The lumbar MRI showed the conus medullaris in a normal position and a fatty filum (FF) extending from L3 to L5, with a maximum diameter of 5.5 mm at L3. A transhiatal approach was performed to section the filum terminale externum (FTE) to achieve spinal cord untethering, and a duplicated FTE was found. The symptoms disappeared immediately but recurred several months later. A second surgical approach was undertaken through a partial sacrectomy via S1-S2 laminectomy, as the section of the FTE did not reveal any other anatomical variants apart from the duplicated FTE, which was sectioned near the inferior part of the dural sac. The symptoms temporarily resolved, but after a few months, they reappeared, presenting differently from the initial symptoms, which involved the proximal parts of the lower limbs. The section of the intradural FF was then performed, and the symptoms disappeared at five years of follow-up. In this case, the potential limitations of the therapeutic capabilities of minimally invasive surgery for section FTE by transhiatal approach, related to the anatomical variation of the duplicate FTE, were eliminated by sectioning the duplicated FTE at the point where the dural sac emerges and by excluding the presence of a duplicate filum terminale internum during the section of the FF. The presence of the FF and its significant thickness should be recognised as a limitation to the effectiveness of the FTE section in treating tethered cord syndrome.

## Introduction

Tethered spinal cord syndrome is a neurological disorder caused by an abnormal stretching of the spinal cord. Clinically, patients with tethered cord syndrome (TCS) display three groups of symptoms, which do not necessarily occur together: progressive back and leg pain, sensorimotor deficits in the lower limbs, and bowel and bladder dysfunction [1,2]. 

Previously, TCS was often diagnosed in children, and in the meantime, adult-onset cases were thought to be rare. In recent years, TCS has been diagnosed in an increasing number of adults. In a recent comprehensive systematic review with more than six thousand cases of TCS in pediatric and adult patients, surgical treatment involved a higher percentage of clinical improvement in adults, but also a higher complication rate in adults [1].

For the treatment of TCS, classic surgery involves sectioning of the filum terminale internum (FTI) under general anaesthesia [1,2]. Recently, a new surgical technique was introduced: the sectioning of the filum terminale externum (FTE). The initial description involved accessing the FTE via sacrectomy under general anaesthesia [3], but later, a minimally invasive technique using local anaesthesia with a transhiatal approach was developed [4,5]. Currently, no time-limiting effects on the effectiveness of FTE have been reported; none of the FTE patients previously documented in the medical literature have exhibited fatty filum (FF).

We report the case of a patient with both FF and FTE.

## Case presentation

A 38-year-old woman presented with persistent low back pain for six years. One year before our assessment, she experienced lumbar trauma. For approximately eight months, she experienced pain and paresthesia resembling an electric shock in the lumbar region and the left lower limb, which caused her left foot to extend onto the ground and led to difficulty walking. 

**Figure 1:**
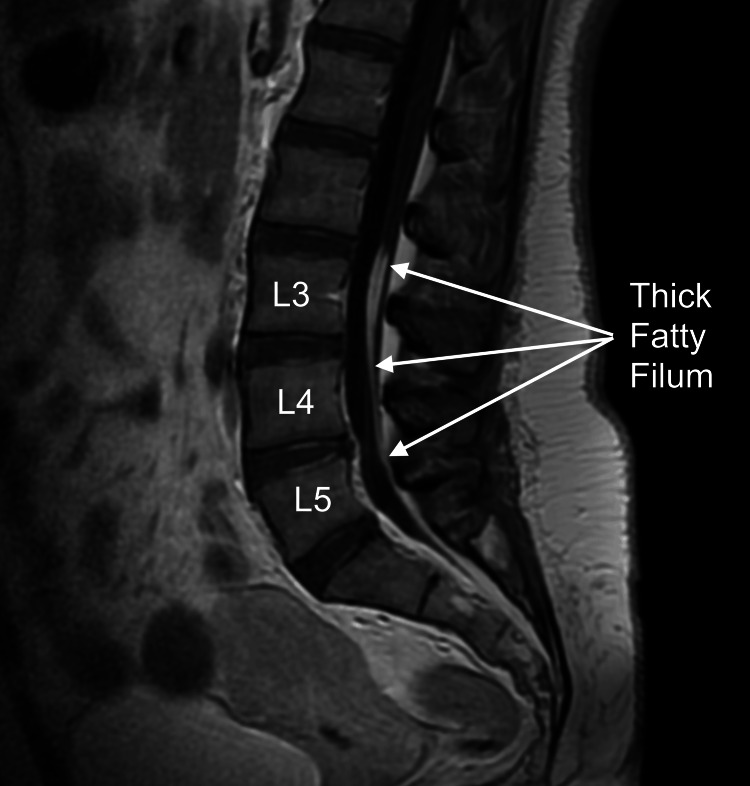
Lumbar magnetic resonance imaging. Sagittal T1-weighted image. Fatty filum extending from L3 to L5, with a maximum diameter of 5.5 mm at L3.

On examination, the patient, in the supine position, could not dorsiflex her left foot due to pain in the lower back and left lower limb; she was unable to raise her left leg, and she could only bend her left thigh if she lay on her left side. She experienced hypo-anaesthesia in the posterior part of the left lower limb. Normal conus medullaris and FF were found on lumbar MR (Figure 1).

A month after her presentation, the patient underwent transhiatal transection of the FTE under local anesthesia with conscious sedation. The sectioning and removal of the superficial dorsal sacrococcygeal ligament provided access to the sacral canal, where the FTE was identified, dissected, and cut using an operating microscope. Deeper within, an abnormal and thin membrane was found inside the sacral canal. This membrane was opened, and another FTE was coagulated and cut. The day after the surgery, the patient showed a dramatic improvement. She was able to walk normally, and there were no limitations in the movements of the left lower limb (Video 1).

**Video 1:** Transhiatal approach preoperative and postoperative assessment

She also experienced no pain in the left lower limb. Her well-being continued for about three months, until, following an abrupt twisting movement of the trunk, lumbago and pain in the left lower limb reappeared, almost as before the surgery. There were no improvements at the follow-up, so a second surgery was performed eight months after the first under general anaesthesia. A partial sacrectomy via S1-S2 laminectomy was carried out, confirming a duplicate FTE and ruling out other anatomical variants that could limit the outcome of the transhiatal approach. The duplicate FTE of equal thickness has been identified up to the inferior part of the dural sac. The duplicate FTE was sectioned (Video 2), and each piece was sent for pathology; no fat tissue was found. The symptoms disappeared immediately after surgery.

**Video 2:** Surgical video Section of duplicate filum terminale externum by partial sacrectomy

The patient remained asymptomatic for a few months after surgery. Then she began to develop symptoms different from her initial presentation, involving both thighs. These symptoms were mild but increased after lifting a weight. The patient walked normally and no longer limped as she did at the onset. She reported tension and stiffness radiating from the lower back to the thighs. While sitting on the bed, she was unable to lift her thighs. However, she did not experience any limitations or pain in plantar flexion and dorsiflexion movements. In a supine position with her legs straight, she could not raise them. The thighs could be rotated laterally, but the movement was reduced on the left side.

Almost two years after the second surgery, the FF section was carried out using a laminectomy approach at L4. There was no duplicate FTI. Immediately in the postoperative assessment, there were no limitations in lower limb movements and no symptoms (Video 3).

**Video 3:** Section of fatty filum preoperative and postoperative assessment

**Figure 2:**
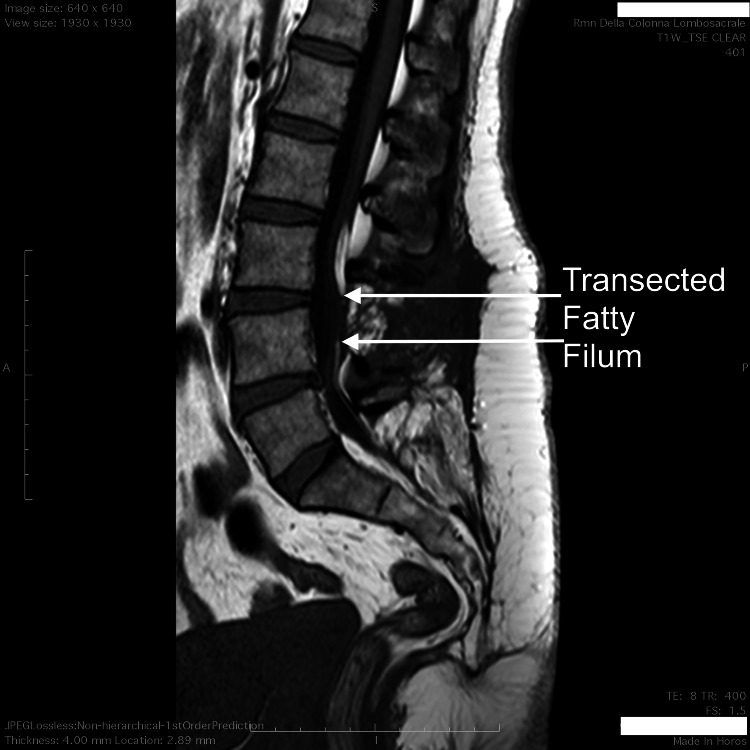
Lumbar MRI after section of the fatty filum. The conus medullaris does not ascend after fatty filum sectioning, but the patient becomes asymptomatic.

Postoperative lumbar MRI revealed the cut of the FF (Figure 2).

At the five-year follow-up, the patient only occasionally experienced low back pain and transient episodes of pain in the left lower limb without any restriction of movement.

## Discussion

Potential complications of traditional surgery by the FTI section under general anaesthesia, including the FF section, are related to the development of permanent intestinal or bladder deficits, new neurological deficits, cerebrospinal fluid leaks, pseudomeningocele, cauda equina tethering, and conus medullaris tethering, which might require additional surgical procedures [1,2]. Wound healing problems and meningitis can also occur, with associated morbidity and mortality. The list is extensive, but the incidence of such complications remains very low. The surgical procedure lasts approximately ninety minutes, and to reduce neurological complications, intraoperative electrophysiological monitoring is recommended, which extends the duration of general anaesthesia. Patients stay in the hospital for two to three days.

When we use minimally invasive surgery with a transhiatal approach in local anaesthesia for the FTE section, the potential complications are limited to wound healing issues. In case of infection, there is no risk of meningitis, with its associated morbidity and mortality, because the meninges are absent at the operative site. The surgery is brief, lasting about 30 minutes, and allows quick patient mobilisation within a few hours. The patient can be discharged on the same day or the next.

A study on fresh-frozen cadaveric specimens concluded that FTE sectioning might not be effective in treating spinal cord untethering in patients with tethered cord syndrome [6]. Veterinary literature reports multiple cases of FTE sectioning in dogs with excellent outcomes [7,8]. One study has shown remarkable results using pre- and post-operative videos [9]. The medical literature reports the sectioning of the FTE in a small group of patients using either the transhiatal [4,5] or sacrectomy [3] approaches. In one case, both the FTE and FTI were sectioned [10]. Currently, no time-limiting effects on the effectiveness of FTE have been reported; none of the patients previously documented in the medical literature have exhibited FF. 

Duplicate FTI is rare, with only six cases documented [11-15]. An additional three cases were identified in medically or spontaneously aborted fetuses [16]. No duplicate FTI associated with a duplicate FTE was observed in our case. Table 1 shows the comparison between the results of this study and other previously published literature on duplicated filum terminale. No patient with a duplicate FTE has been reported in the literature, and no duplicate FTE was found in the anatomical studies of cadavers [17-21]. In our personal experience, this is the only case of a duplicate FTE in the more than 400 patients we have operated on (unpublished data). 

**Table 1:** Reported cases of duplicated filum terminale with or without tethered cord syndrome FF: fatty filum; SCM: split cord malformation; FTI: filum terminale internum; FTE: filum terminale externum; M: male; F: female; NM: not mentioned; NA: not applicable

Author/	Age years	Sex	Symptoms	FF	Level of Conus	SCM	Surgery	Outcome
Year								
Current Case	38	F	Patient limps due to paroxysmal pain in her left leg. Numbness and hypoesthesia in her left leg.	Yes (no duplicate FTI)	Upper part of L2	No	Sectioning of duplicate FTE	Symptoms disappeared
			Recurrence of symptoms with tension and stiffness radiating from her lower back to both thighs, but her gait is normal.				Reoperation for sectioning of FF	Symptoms disappeared
Xu et al., 2020 [12]	45	F	Numbness in both legs and paroxysmal leg pain	No	L5-S1 disk	Yes	Sectioning of duplicate FTI	Symptoms disappeared
Milic et al., 2019 [11]	3	F	Spondylocostal dysostosis, gate disturbance, urinary incontinence	No	L4	Yes	Duplicate FTI. Her parents refused a surgical intervention	-
Davanzo et al., 2016 [14]	43	M	Lower back pain, atrophy of left calf	No	L5-S1 disk	No	Sectioning of duplicate FTI. Reoperation for repair pesudomeningocele	Significantly improved
Starnoni et al., 2016 [15]	47	M	Lower back and leg pain, gait unsteadiness	One of the duplicate FTI	L3-4 disk	No	Sectioning of duplicate FTI	Improved
Rizk et al., 2014 [13]	8	NM	Asymptomatic	One of the duplicate FTI	NM	No	Sectioning of duplicate FTI	NM
	5	NM	Asymptomatic	Both duplicate FTI	NM	No	Sectioning of duplicate FTI	NM
Salbacak et al., 2000 [16]	NA	NM	NM (aborted fetus)	-	NM	NM	-	-
	NA	NM	NM (aborted fetus)	-	NM	NM	-	-
	NA	NM	NM (aborted fetus)	-	NM	NM	-	-

In this case, two approaches were adopted for the FTE section to exclude other anomalies or divisions at the level of the FTE emergence from the dural sac that could not be assessed with the initial transhiatal approach. Still, no anatomical variants other than the duplication were identified. The initial clinical picture, characterised by symptoms distal to the left lower limb with significant difficulty walking, disappeared after the FTE section. Subsequently, the residual subclinical spinal cord traction later became clinically evident when there was a sudden traction of the spinal cord caused by the abrupt twisting of the spinal canal or after lifting a weight. When the symptoms returned, they were located proximal to the lower limbs, but this was accompanied by regular walking. In this case, the section of the FTE, in the presence of a very thick FF, did not allow for adequate release of the spinal cord to achieve definitive healing.

## Conclusions

In this case, the potential limitations of the therapeutic capabilities of minimally invasive surgery for section FTE by transhiatal approach, related to the anatomical variation of the duplicate FTE, were eliminated by sectioning the duplicated FTE at the point where the dural sac emerges and by excluding the presence of a duplicate FTI during the section of the FF. The presence of the FF with significant thickness and extending from L3 to L5 is a limitation to the effectiveness of the FTE section in treating TCS.
